# Genomic signatures of selection in response to sulfate air pollution in natural populations of red spruce

**DOI:** 10.1186/1753-6561-5-S7-O5

**Published:** 2011-09-13

**Authors:** Om Rajora, Stanislav Bashalkhanov

**Affiliations:** 1 Faculty of Forestry and Environmental Management, University of New Brunswick, 28 Dineen Drive, Fredericton, NB E3B 5A3, Canada

## 

Rapid environmental changes, such as anthropogenic air pollution, can create significant evolutionary pressures on populations, species and ecosystems. Evolutionary processes occurring in natural populations at very short time scales, especially in response to human-induced environmental changes, are not well understood. Confounding effects of geographic variation and demography cannot be easily separated from signatures of recent selection in natural populations. We investigated the genetic response of declining red spruce (*Picea rubens*) populations at high elevation sites in Southern Appalachians to anthropogenic sulfate depositions. Red spruce seedlings are more sensitive to drought and cold stresses elicited by exposure to anthropogenic sulphate air pollution, than old trees. Genetic variation in seedlings and young trees was significantly reduced in heavily polluted stands. Several candidate genes involved in cold acclimation and calcium metabolism demonstrated signatures of selection corresponding with sulfate pollution levels. SNP allele frequencies at one gene involved in calcium metabolism demonstrated directional selection in response to anthropogenic sulfate deposition in red spruce growing at severely polluted high elevation sites, which corresponds well with the putative role of this gene in adaptation to acidification stress. Unlike range-wide experimental designs (e.g. the popular F*_ST_* outlier test) and nucleotide diversity-based association studies, our within-population testing approach disentangled the confounding effects of geographic variation and demography from the genetic effects of recent selection.

